# Identification and validation of prognostic signature genes of bladder cancer by integrating methylation and transcriptomic analysis

**DOI:** 10.1038/s41598-023-50740-x

**Published:** 2024-01-03

**Authors:** Dipankor Chatterjee, Sadia Islam Mou, Tamanna Sultana, Md. Ismail Hosen, Md. Omar Faruk

**Affiliations:** Department of Biochemistry and Molecular Biology, University of Dhaka, Dhaka, 1000 Bangladesh

**Keywords:** Cancer, Computational biology and bioinformatics, Biomarkers, Molecular medicine, Oncology

## Abstract

Being a frequent malignant tumor of the genitourinary system, Bladder Urothelial Carcinoma (BLCA) has a poor prognosis. This study focused on identifying and validating prognostic biomarkers utilizing methylation, transcriptomics, and clinical data from The Cancer Genome Atlas Bladder Urothelial Carcinoma (TCGA BLCA) cohort. The impact of altered differentially methylated hallmark pathway genes was subjected to clustering analysis to observe changes in the transcriptional landscape on BLCA patients and identify two subtypes of patients from the TCGA BLCA population where Subtype 2 was associated with the worst prognosis with a *p*-value of 0.00032. Differential expression and enrichment analysis showed that subtype 2 was enriched in immune-responsive and cancer-progressive pathways, whereas subtype 1 was enriched in biosynthetic pathways. Following, regression and network analyses revealed Epidermal Growth Factor Receptor (EGFR), Fos-related antigen 1 (FOSL1), Nuclear Factor Erythroid 2 (NFE2), ADP-ribosylation factor-like protein 4D (ARL4D), SH3 domain containing ring finger 2 (SH3RF2), and Cadherin 3 (CDH3) genes to be the most significant prognostic gene markers. These genes were used to construct a risk model that separated the BLCA patients into high and low-risk groups. The risk model was also validated in an external dataset by performing survival analysis between high and low-risk groups with a *p*-value < 0.001 and the result showed the high group was significantly associated with poor prognosis compared to the low group. Single-cell analyses revealed the elevated level of these genes in the tumor microenvironment and associated with immune response. High-grade patients also tend to have a high expression of these genes compared to low-grade patients. In conclusion, this research developed a six-gene signature that is pertinent to the prediction of overall survival (OS) and might contribute to the advancement of precision medicine in the management of bladder cancer.

## Introduction

With a tenfold higher risk of occurrence compared to women, bladder cancer (BLCA) is the tenth most prevalent cancer worldwide and the sixth most prevalent in men^1^. Early in the prognosis, muscle-invasive or metastatic cancer is identified in around 25% of BLCA patients^2^. Patients with non-muscle-invasive BLCA are nevertheless experiencing significant rates of progression. Generally, the 5-year survival rate for bladder cancer is less than 20% at all stages. Even though there are multiple drugs, the lack of clinically potential biomarkers hinders the development of optimal treatment^3^. Tumor recurrence and metastasis are two key risk factors that have a significant impact on patients with BLCA prognosis. Thus, research concentrating on early identification and the prevention of the growth and spread of BLCA must be carried out with the help of efficient diagnostic procedures including optical techniques, imaging systems, and tumor biomarkers, to enhance the survival and reduce recurrence and progression of BLCA patients^4,5^.

Cystoscopy is a routine operation almost everywhere due to its high sensitivity and is still the gold standard for BLCA, but because of its invasiveness, it has adverse effects^6^. Nevertheless, urine cytology has poorer sensitivity for low-grade cancers even if it is a wonderful method for high-grade malignancies^7^. In this case, biomarkers are crucial and many biomarkers have been investigated during the past few years in a variety of clinical scenarios, including screening, monitoring, and follow-up^8^.

Urine cytology is currently the recommended urine marker for bladder cancer detection, but its sensitivity for detecting low-grade tumors is limited^9^. Single protein-based markers like NMP22 and BTA may be influenced by benign conditions, leading to false-positive results^10^. In recent years, commercially available markers have focused on multiplex protein, mRNA, and DNA assays. Non-coding RNA forms and extracellular vesicles are emerging areas of research for novel urinary biomarkers^11^. Genomic markers provide molecular insights into tumor biology and have potential applications beyond diagnosis and surveillance. Although non-invasive assays for bladder cancer (BC) diagnosis and follow-up currently exhibit high sensitivities and specificities, they still entail inconvenient rates of false positive results. Therefore, the development of more advanced and specific prognostic biomarkers holds promise for improving bladder cancer detection and management^12^.

The investigation of prognostic biomarkers in BLCA patients is still quite novel. Often, while constructing gene sets, cells or immunological pathways are the targets. Hallmark pathway-related gene sets and particular molecular pathway-selected gene sets have recently gained a lot of popularity in this area of research^13,14^. Typically, the research designs of these studies revolve around investigating immune cells and their impact on prognosis. Differential expression and route enrichment analyses are used in several studies^15,16^. Predictive modeling has become increasingly prevalent as a result of the creation of a prognostic gene set to validate them^17^.

With the development of molecular biology and bioinformatics over the past few decades, a number of unique bladder cancer tumor biomarkers have been found. Despite this advancement, key BLCA risk profiles have been developed using genes associated with a variety of symptoms. Potential machine learning algorithms are now being developed to group patients based on their clinical, molecular markers and prognostic factors^18^. To yet, little research has been done on the precise characterization of prognostic biomarkers associated with hallmark pathways^19^.

DNA methylation is a significant epigenetic alteration that has a significant impact on the transcriptional regulation of gene expression. In studies on tumors, it has been discovered that altering DNA methylation results in abnormalities in gene structure and function, which can serve as an early indicator of the development of tumors^20^. Additionally, many studies have been undertaken to understand the epigenetic mechanism behind cancer progression and resistance^21,22^. Hence, this novel study concentrated on the utilization of methylation data to study the impact of altered methylation pattern in bladder cancer patients by exploiting transcriptome analysis, and clinical traits.

Initially, methylation and survival analysis revealed prognostic hallmark genes and subjected them to clustering of the BLCA patients. Following transcriptomic analysis between two clusters identified novel biomarkers that may be involved in the survivability of bladder cancer patients and validated using the TCGA database and external dataset. The study proposed prognostic signature genes that can predict a patient’s prognosis and also validated using an external dataset. The signature genes were found to be associated with immune cells which may help in immune therapy. Collectively, the study followed a unique approach by integrating methylation, transcriptomic, and clinical data analysis to identify such prognostic biomarkers and was therefore provided with a novel insight to investigate bladder cancer.

## Method

### Data acquisition

This study utilized RNASeq, methylation, and clinical data of bladder carcinoma (BLCA) from The Cancer Genome Atlas (TCGA) to uncover prognostic biomarkers (https://www.cancer.gov/ccg/research/genome-sequencing/tcga). Gene sets of cancer hallmark pathways were collected from the Molecular Signatures Database v7.x (MSigDB; https://www.gsea-msigdb.org/gsea/msigdb), which were about 2322 genes. The study included 406 bladder cancer patients and 19 normal samples as training data and the GSE13507 dataset was used as test data. UCSC Xena the University of California, Santa Cruz (UCSC), Xena Browser (https://xenabrowser.net/) was used to download transcriptomic data of the TCGA which were already preprocessed^23^.

### Differential methylation analysis

Methylation data were downloaded from the TCGA of patients as well as normal samples. Filtering was performed by removing CpG probes corresponding to chromosome X and chromosome Y. Overlapping probes were also filtered out to uniquely identify the differentially methylated genes. The data were downloaded as beta values which were then converted to M-value by executing log2 (beta/1-beta) transformation as M-value is easier to interpret, where positive M-value indicates hypermethylated and negative value indicates down methylation^24,25^. The limma package was used for differential methylation analysis between normal and bladder cancer patients using R programming language^26^. Cut-off criteria for adjusted value and |log fold change| were < 0.05 and > 1.5 respectively.

### Clustering, identification, and characterization of molecular subtypes

Significantly methylated genes were then subjected to Cox regression survival analysis to identify the prognostic genes involved in the survivability of the cancer patient by considering *p*-value < 0.05. Hallmark pathway genes were then extracted from the significant prognostic genes, which were then considered to cluster the TCGA bladder cancer patients by unsupervised non-negative matrix factorization (NMF) clustering method using R package NMF^27^. The operation was performed by setting the method to “Brunet” and nrun to 50. This test was performed for ranks 2–6 in order to find the best cluster rank. The clustering performance was assessed by the principal components analysis (PCA) of the transcriptome data of TCGA BLCA. PCA plot was drawn considering K-means clustering using the Fviz_cluster method in R^28,29^. Overall survival (OS) and disease-free survival (DFS) analysis was performed using the survival package in R and cancer tumor stage, neoplasm histologic grade, neoplasm disease stage and metastasis stage were also observed between these subtypes using cBioPortal^30^.

### Assessing amplification and deletion of chromosome between subtypes

To assess the genomic instability between subtypes Genomic Identification of Significant Targets in Cancer (GISTIC) v2.0 was used to observe the amplification and deletion in different chromosomes between subtypes^31^. Amplification and deletion data were downloaded from cBioPortal database based on the subtype of TCGA BLCA population and subjected to GISTIC server for CNV analysis.

### Immunological assessment between subtypes

The number of stromal and immune cells infiltrating BLCA tissues was compared between subtypes by utilizing the ESTIMATE technique developed by the National Institute of Health. Estimate score was calculated for each sample considering both stromal and immune scores which indicate the tumor purity within each sample^32^. Subsequently, the CIBERSORT method was exploited to determine the strength of 22 immune cell types in each TCGA BLCA sample. CIBERSORT employs a deconvolution criterion via linear support vector regression (SVR) to deconvolute the gene expression profile^33^. The Wilcoxon rank-sum significance test was performed between subtypes to understand the difference in immune filtration levels^34^. The data for CIBERSORT analysis was extracted from the CIBERSORT website and the UCSC Xena browser.

### Differential expression and enrichment analysis between subtypes

Differential expression data between molecular subtypes was collected from the cBioPortal website and a set of cutoff values (False Discovery Rate: FDR < 0.05 and |log2fold|> 1) was employed to determine the differentially expressed genes (DEGs). The upregulated genes of molecular subtypes were then utilized for enrichment analysis including biological process and KEGG: Kyoto Encyclopedia of Genes and Genomes, analysis via Enrichr webserver^35–37^. R programming was used to perform the analysis.

### Identification of signature gene set

DEGs were subjected to a univariate Cox proportional hazards regression analysis considering the survival data of the BLCA cancer patients. For this, “coxph” and “survival” packages were utilized in R with an adjusted *p-*value < 0.05 as the cut-off value^38,39^. In addition, the least absolute shrinkage and selection operator (Lasso) method was employed on the resultant data from univariate analysis to create a predictive model with a high degree of accuracy. Lasso regression narrows the coefficients of the remaining variables toward zero and only chooses a subset of the important predictor variables. The penalty term employed by Lasso encourages the coefficients of the less important variables to be shrunk toward zero, effectively removing them from the model^40,41^. The resulting model is simpler and more interpretable, with a reduced risk of overfitting and multicollinearity problems. For the analysis, survival time and survival status were considered as independent variable, where the genes were dependent variable. The result reveal significant genes that influence patients survival.

### Risk score and risk model construction

Following lasso regression analysis, multivariate analysis was performed on the significant genes from the lasso model with a cut-off *p*-value of < 0.05 in order to identify the most significant prognostic markers. The resultant genes were then subjected to network analysis and enrichment analysis to observe the involvement in cancer progression by NetworkAnalyst^42^. Network analysis involves constructing and visualizing biological networks to understand gene interactions. From the network analysis, best hub genes were considered for final prognostic gene markers, and a risk formula was constructed utilizing regression coefficient and RNAseq expression data of the final gene set^43^. The formula goes by,

$$\begin{aligned} {\mathbf{Risk}} \, {\mathbf{Score}} = & {\text{Coefficient1}}*{\text{expGene1}} + {\text{Coefficient2}}*{\text{expGene2}} + {\text{Coefficient3}}*{\text{expGene3}} \\ & + {\text{Coefficient4}}*{\text{expGene4}} + {\text{Coefficient5}}*{\text{expGene5}} + {\text{Coefficient6}}*{\text{expGene6}} \\ \end{aligned}$$ where expGene1 to expGene6 represented the gene expression levels which were continuous values of the identified genes for a particular patient and coefficient 1 to coefficient 6 are the corresponding regression coefficients representing the impact of each gene's expression on the overall risk score.

Using the risk scores the BLCA patients were separated into two groups including high-risk and low-risk groups based on the median value. The survival analysis was performed between these two groups using the survival package in R and observed the Kaplan–Meir curves for overall survival^44^. Receiving operating characteristic curves (ROCs) were generated for 1 year, 3 years, and 5 years OS and AUC values were calculated to examine the prediction potential of the risk model using the timeROC package in R. The AUC value measures the overall performance of a binary classification model and provides an intuitive way to compare different models^45,46^. The timeROC package provides resources for studying time-dependent ROC curves in survival analysis. It provides a simple interface for computing, analyzing, and comparing the prediction accuracy of numerous biomarkers or tests across time. The expression of the final gene set was also observed in a heatmap and the independence of the risk score for OS was examined using multivariate Cox regression analysis using clinical factors. The survminer package was then used to create a forest plot that included the hazard ratio (HR) and 95% confidence interval (CI) for each variable^47^.

### Nomogram model generation

Using the rms package in R, a nomogram for predicting overall survival (OS) and disease-free survival (DFS) was created by combining clinical data and the risk score of the TCGA BLCA patients. Based on the regression coefficients of the individual variables, scores were assigned. The matching individual scores of all factors were added up to provide a total score for each patient. Following that, the likelihood of each patient's outcome was determined using the conversion function. Calibration plots were used to examine the nomogram's prediction capability with a default penalty factor = adapen and boot. times = 10 which indicates an internal sampling for the test. As for model selection, “lasso” was used with an nfold of 5 which specifies the number of folds or subsets into which the dataset will be divided during the cross-validation process^48^.

### Validation of the signature gene set in an independent dataset

To further assess the robustness of the signature gene set, an external dataset was used to validate the result. GEO dataset GSE13507^49^ was used with 165 primary cancer patients and the data was split into high and low-risk groups based on the threshold value of the risk scores calculated by the risk formula. Kaplan–Meir curves were observed for overall survival and AUC values were calculated using the timeROC package in R, where the time unit was considered in the year.

### Correlation analysis between signature gene set and interleukins as well as immune cell filtration

The correlation between gene set expression with interleukin-6 (IL-6) and interleukin-20 (IL-20) was examined using the GEPIA2 web server^50^. Both IL-6 and IL-20 have a significant association with bladder cancer progression and metastasis^51–53^. With the help of the TCGA BLCA cohort, the UALCAN server was utilized to monitor the expression of genes at different phases of BLCA cancer patients^54^. The relationship between gene expressions and tumor-infiltrating immune cells (TIICs), including B-cells, T-cells, macrophages, neutrophils, and others, was observed using the TIMER server^55^. In order to calculate the frequency of TIICs from gene expression patterns, the server considered available TCGA cohort data. The expression pattern of the signature gene set was also observed in different stages and grades of bladder cancer.

### Mutational assessment between subtypes

cBioPortal server was used to observe the mutational status of the 6 signature genes in subtype 1 and subtype 2. Missense mutations, inframe mutations, truncating mutations, and others were compared between subtypes to comprehend the impact of mutation in regulating the expressions of these genes.

### Single-cell RNA-seq and grade expression analyses

The analysis of single-cell RNA sequencing data was conducted using the Tumor Immune Single-cell Hub 2 web service^56^, accessible at http://tisch.comp-genomics.org/home/. The study employed the uniform manifold approximation and projection (UMAP) method to reduce data dimensionality and visualize the clustering outcomes. Additionally, mRNA expression patterns of distinct cells were illustrated using UMAP distribution Figures. GSE130001^57^ and GSE149652^58^ bladder cancer datasets were utilized to observe the expression of pattern of the signature genes in different cell types within tumor microenvironment and intrtumor immune cells, respectively. Expression pattern was also observed between high and low grade patients for the signature genes using TCGA BLCA FPKM count data.

## Result

**Figure 1:**
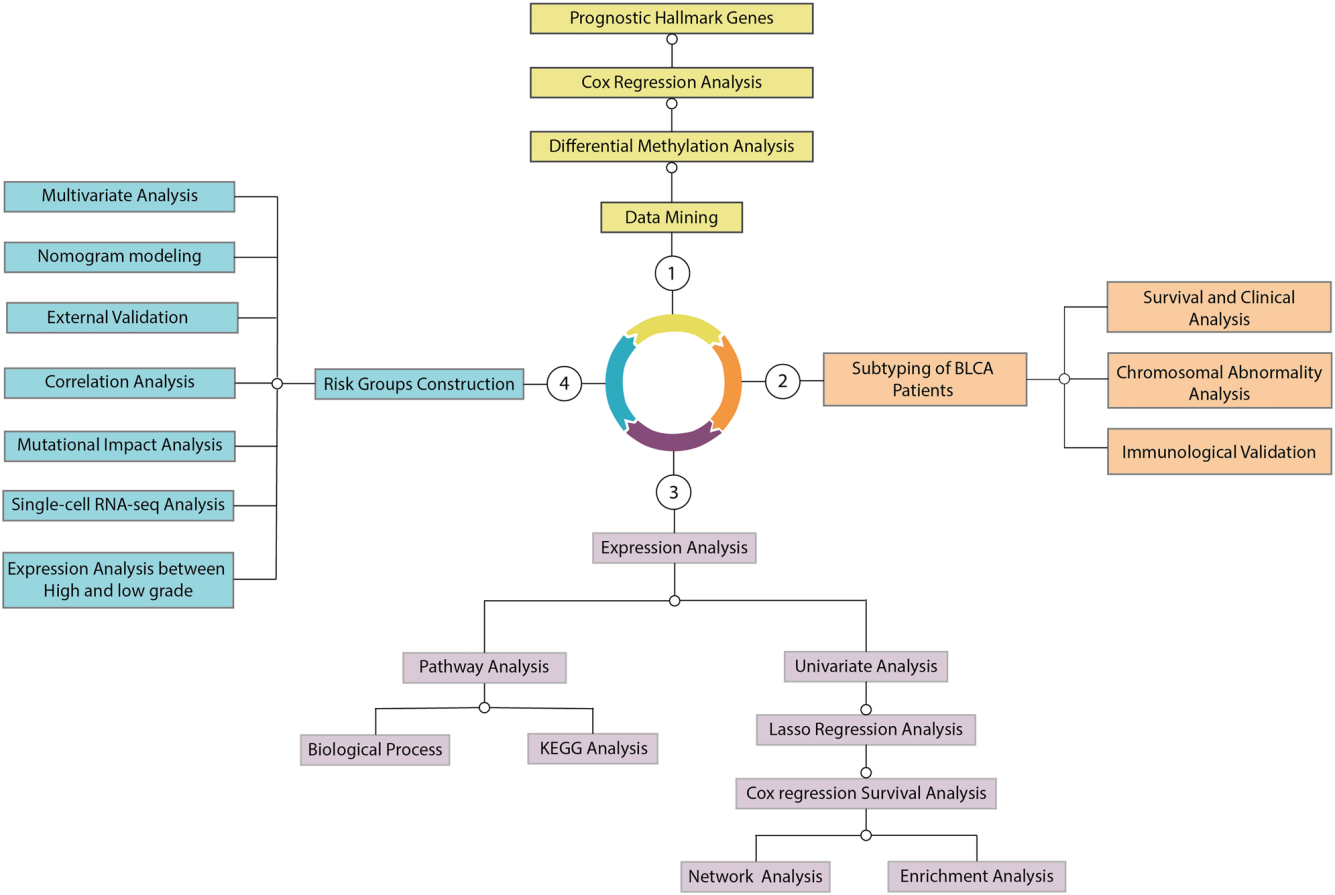
Workflow of the experiment. The workflow was structured into four sequential Sects. (1, 2, 3, and 4), each denoted by the (-o) sign to signify the analysis direction. The outcomes generated from each section were subsequently utilized, following a sequential flow from section "Introduction" to section "Method", then from section "Method" to section "Result", and finally from section "Result" to section "Discussion".

The workflow of the study is summarized in Fig. 1. All the tools and algorithms used in this study were represented in Supplementary Table 1.

### Identification of differentially methylated hallmark genes

**Figure 2:**
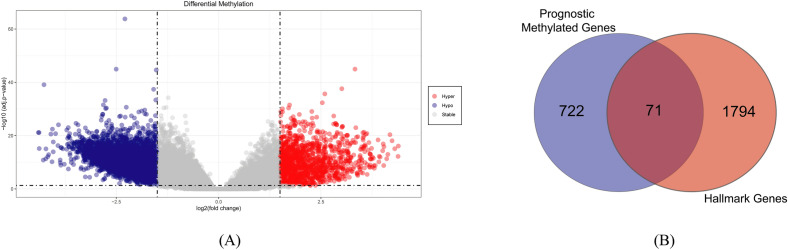
(**A**) Volcano Plot visualization for differential methylation where red color indicated hyper methylated genes and blue color meant hypo methylated gene. |LogFC|> 1.5 and p-value < 0.05 were employed to identify significant results (**B**) Venn diagram of hallmark genes identification.

Methylation analysis between normal and bladder cancer patients revealed 3606 differentially methylated genes (Fig. 2A), which were then subjected to Cox regression analysis to identify prognostic genes and the analysis uncovered 793 genes associated with the survival of the bladder patients. Following this analysis, 71 genes of hallmark pathways were identified from these prognostic genes and subjected to the clustering of the bladder cancer patients (Fig. 2B). These genes were than utilized to observe the impact of altered methylation pattern in TCGA BLCA population in terms of transcriptional landscape.

### Creation of molecular subtypes

**Figure 3:**
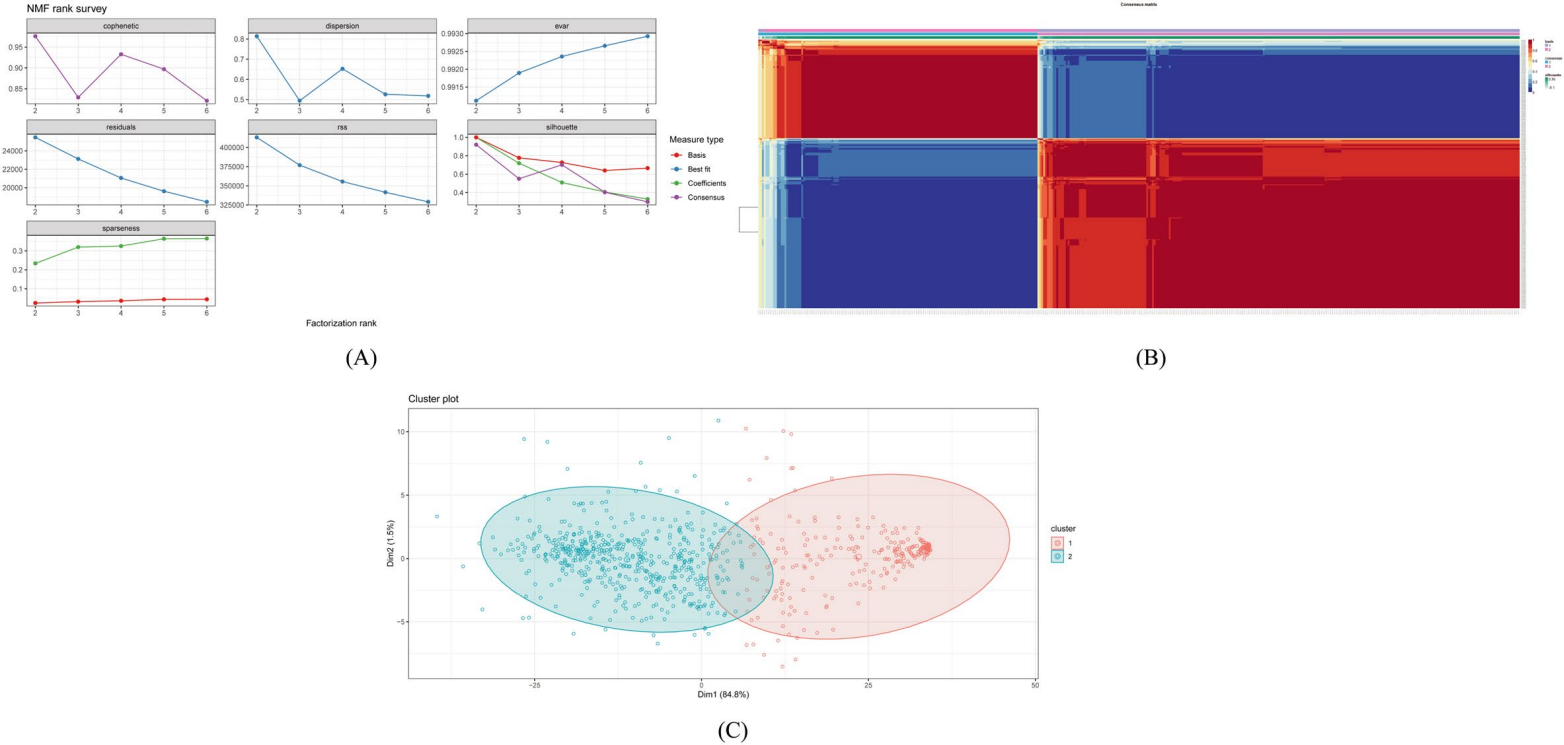
Clustering of TCGA BLCA patients. (**A**) Ranking of factors for k = 2–6. Less than two cophenetic and rss values indicate that two clusters are the most equally dispersed throughout the samples. (**B**) Heat diagram of the consensus matrix at k = 2. The values are between 0 and 1. Using hierarchical clustering, the columns and rows are arranged according to the average link's Euclidean distance. The samples are organized equally when k = 2, or two clusters, which denotes an ideal clustering number. (**C**) Visualization of the cluster using PCA plot.

NMF clustering algorithm was employed on the expression profile of the selected hallmark genes of the cancer patients (n = 406) via NMF package in R. As for method parameter “Brunet” was used with a rank of 2:6 and nrun was 50. Based on the cophenetic correlation coefficient, the best rank was selected for clustering which was k = 2 as the cophenetic began to fall from 2 (Fig. 3A). When k = 2, the heatmap naturally displayed the consensus matrix (Fig. 3B). A consensus map for all 2 to 6 ranks was visualized and rank 2 showed the best clustering (Supplementary Fig. 1). The PCA analysis also supported the clustering into two subtypes (Fig. 3C) and therefore, the BLCA samples were divided into subtype 1 (n = 250) and subtype 2 (n = 156) molecular subtypes.

### Validation and characterization of molecular subtypes

**Figure 4:**
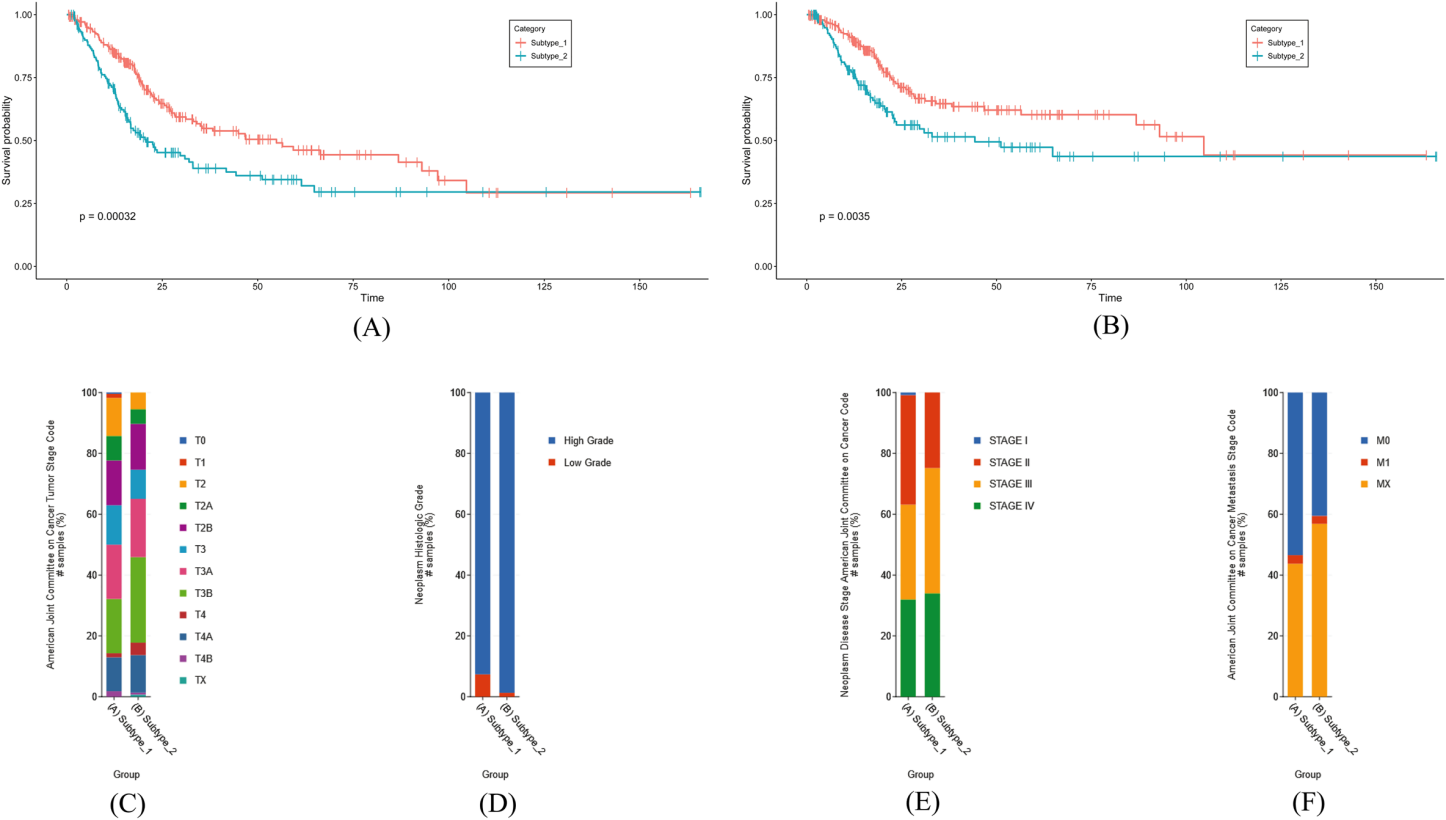
Kaplan–Meier (**A**) overall survival plot and (**B**) Disease-free survival plot observation between two subtypes and *p*-value was calculated to measyre the significance of the analyses. Comparison of (**C**) tumor stage, (**D**) neoplasm grade, (**E**) neoplasm disease stage, and (**F**) metastasis stage between two clusters. The colors indicated distinct characteristic group of patients.

Clinical evaluation was conducted for two subtypes among patients diagnosed with BLCA (Bladder Cancer). Overall survival and disease-specific survival analyses revealed that subtype 2 significantly displayed the worst prognosis than subtype 1 (Fig. 4A,B). Clinical assessment was done between two subtypes by observing tumor stage, neoplasm histologic grade, neoplasm disease stage, and metastasis stage. In each case, subtype 2 involved patients with higher percentage of T4 invasive, high grade, and stage 4 indicating the worst clinical outcome compared to subtype 1 (Fig. 4C–F).

### Chromosomal amplification and deletion analysis between two subtypes

The mutation frequencies of CNV were observed for two subtypes of bladder cancer patients. The most frequently amplified chromosome for both subtypes was observed in chromosomes 8 and 20. On the other hand, chromosomes 9 were found to be the most frequently deleted chromosome in subtype 1 and chromosome 22 were most frequently deleted in subtype 2. Overall, the mean frequency of amplification of subtype 2 (0.2689120) was lower than subtype 1 (0.2736826) and the mean deletion for subtype 2 (0.2864311) was higher than subtype 1 (0.2729289) (Supplementary Fig. 2).

### Stromal and immune cells estimation in bladder tumors

Focusing on the stromal and immune cells that make up the bulk of the non-tumor components in tumor samples, the stromal and immunological assessment was performed by looking for distinctive signatures connected to stromal and immune cell infiltration in tumor tissues. The ESTIMATE score is based on these to infer tumor purity in tumor tissues. The poor prognosis subtype 2 was indicated by high levels of the stromal score, immunological score, and ESTIMATE score (Supplementary Fig. 3A), which validated the survival analysis. The *P*-value was calculated using the Wilcoxon rank test and was found to be significant for each scoring analysis (*p*-value < 0.05).

### Tumor purity comparison using multiple methods

Five different methods—ESTIMATE, ABSOLUTE, LUMP, IHC, and CPE—were used to assess tumor purity. All of the data demonstrated a strong distinction between the subtypes in the tumor purity score, with subtype 2 exhibiting lower tumor purity and a worse prognosis (Supplementary Fig. 3B). Based on the Wilcoxon rank test, the *p*-value was calculated, which were all statistically significant (*p*-value < 0.05).

### Immune checkpoint gene assessment

**Figure 5:**
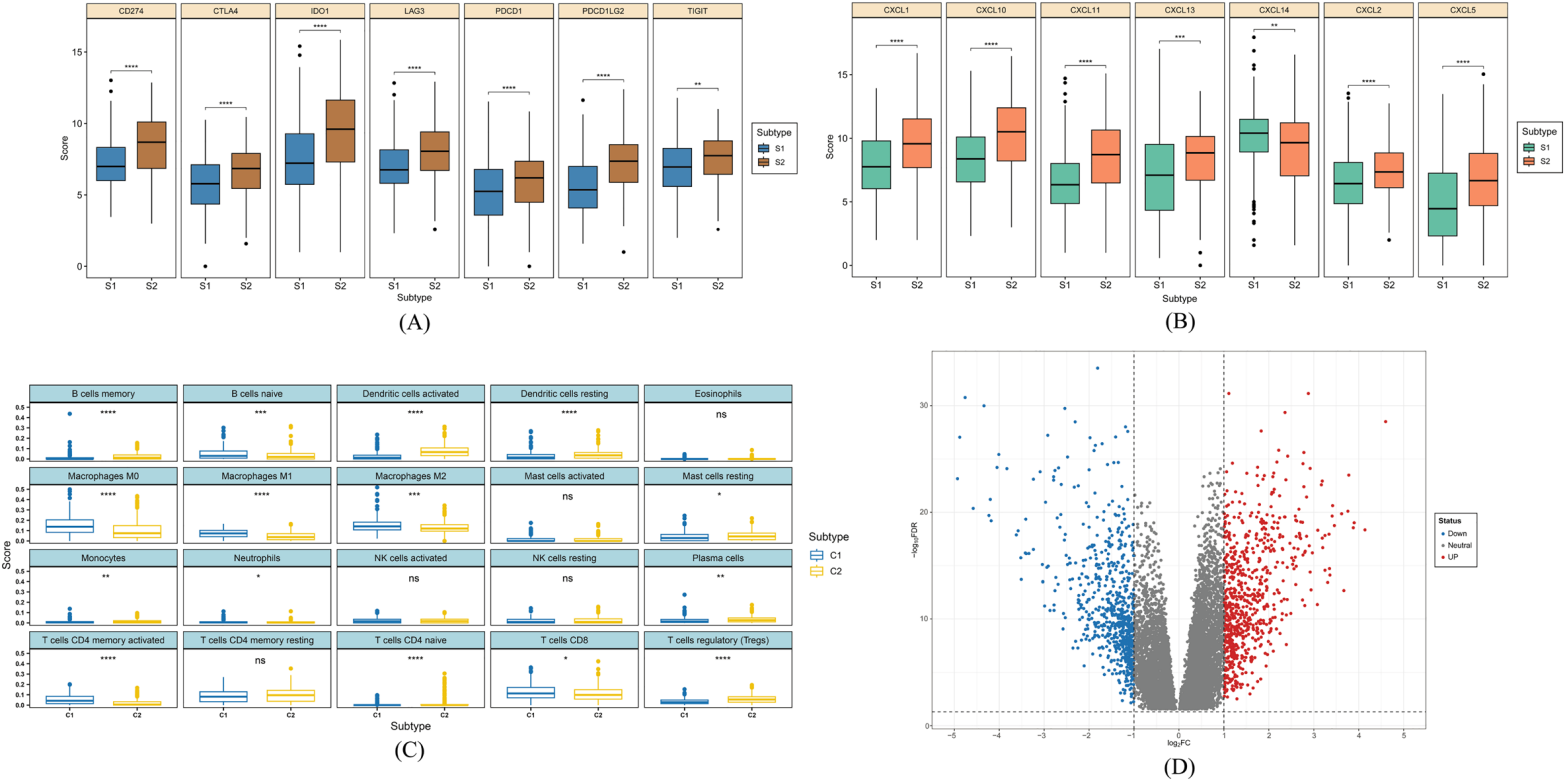
Immunological analysis of (**A**) immune checkpoint genes, (**B**) chemokines, and tumor-infiltrating cells between two clusters and p-value < 0.05 was considered to be significant. Differentially expressed genes between two clusters observation through (**D**) volcano plot by employing |logFC|> 1 and FDR < 0.05 parameters.

LAG3 (Lymphocyte-activation gene 3), PDCD1LG2 (Programmed cell death 1 ligand 2), CD274 (cluster of differentiation 274), IDO1 (indoleamine 2,3-dioxygenase 1), PDCD1 (Programmed Cell Death 1), CTLA4 (Cytotoxic T-lymphocyte-associated protein 4), and TIGIT (T cell immunoreceptor with Ig and ITIM domains) were considered important immune checkpoint genes because these genes are activated when T lymphocytes recognize and connect to related proteins on other cells, such as certain tumor cells. The T cells are informed that the checkpoint and partner proteins are "off" whenever they interact. This can make it more difficult for the immune system to get rid of cancer. All of these genes were elevated in subtype 2, and the Wilcoxon rank test revealed statistical significance for each of them (Fig. 5A). These findings demonstrate conclusively the potential importance of subtyping.

### Chemokines expression observation

Across the two subtypes, CXC chemokine expression levels were compared. Analysis was done on the chemokines connected to bladder cancer using their expression profile from TCGA data. CXCL1, CXCL2, CXCL5, CXCL10, CXCL11, and CXCL13 all exhibited greater expression of Subtype 2, whereas CXCL14 had a higher expression of the subtype 1 (Fig. 5B). In bladder cancer, CXCL14 naturally plays a protective effect, supporting earlier findings ^59^. Other chemokines are often associated with a poorer prognosis for BLCA, supporting earlier research.

### Infiltration level analysis between two subtypes

Analysis of 22 TICs was done to find out more about the connections between the subtypes and the immunological milieu since the differences between the two groups were connected to immunity. The 22 TIC profiles required for the ensuing experiments were first created using the CIBERSORT technique. Following that, the outcomes for the two subtypes were compared. The quantity of each cell component differed noticeably between the two subtypes (Fig. 5C). All the details are provided in Supplementary Table 2.

### Expressional and ontology analysis between subtypes

Expression analysis between subtypes revealed 1430 significantly differentially expressed genes (DEGs), where 730 and 700 genes were found to be upregulated in Subtype 1 and Subtype 2, respectively (Fig. 5D). These upregulated genes were further assessed by enrichment analysis. This analysis was done for KEGG and the biological process. Whereas the immune system-related pathways were concentrated in the upregulated genes of subtype 2, those of subtype 1 were mostly involved in steroid hormone production and metabolic pathways (Supplementary Fig. 4A–B). The pathways for the Cytokine-Cytokine Receptor, Toll-Like Receptor Signaling, and cancer-related pathways exhibited high enrichment scores and significance for the subtype 2 elevated genes (Supplementary Fig. 4D). Major enriched pathways associated with cytochrome P450, hormone, lipid synthesis, etc. were associated with the Subtype 1 upregulated genes (Supplementary Fig. 4C). For this analysis, FDR had a cutoff threshold value of 0.05.

### Univariate and Lasso regression analysis of DEGs

The DEGs of Subtype 1 versus Subtype 2 were subjected to univariate cox regression analysis to uncover prognostic genes. A total of 405 genes were found to be significant from the analysis with an FDR < 0.05. Following univariate analysis, lasso regression analysis was performed on these genes to identified genes with significant prognostic potential and the analysis revealed 50 genes after applying minimum lambda value. These genes were further analyzed for survivability of the patients by considering survival status and survival time which lead to the determination of 16 most significant genes (Supplementary Fig. 5A,B).

### Cox regression analysis of 50 prognostic genes and network analysis

**Figure 6:**
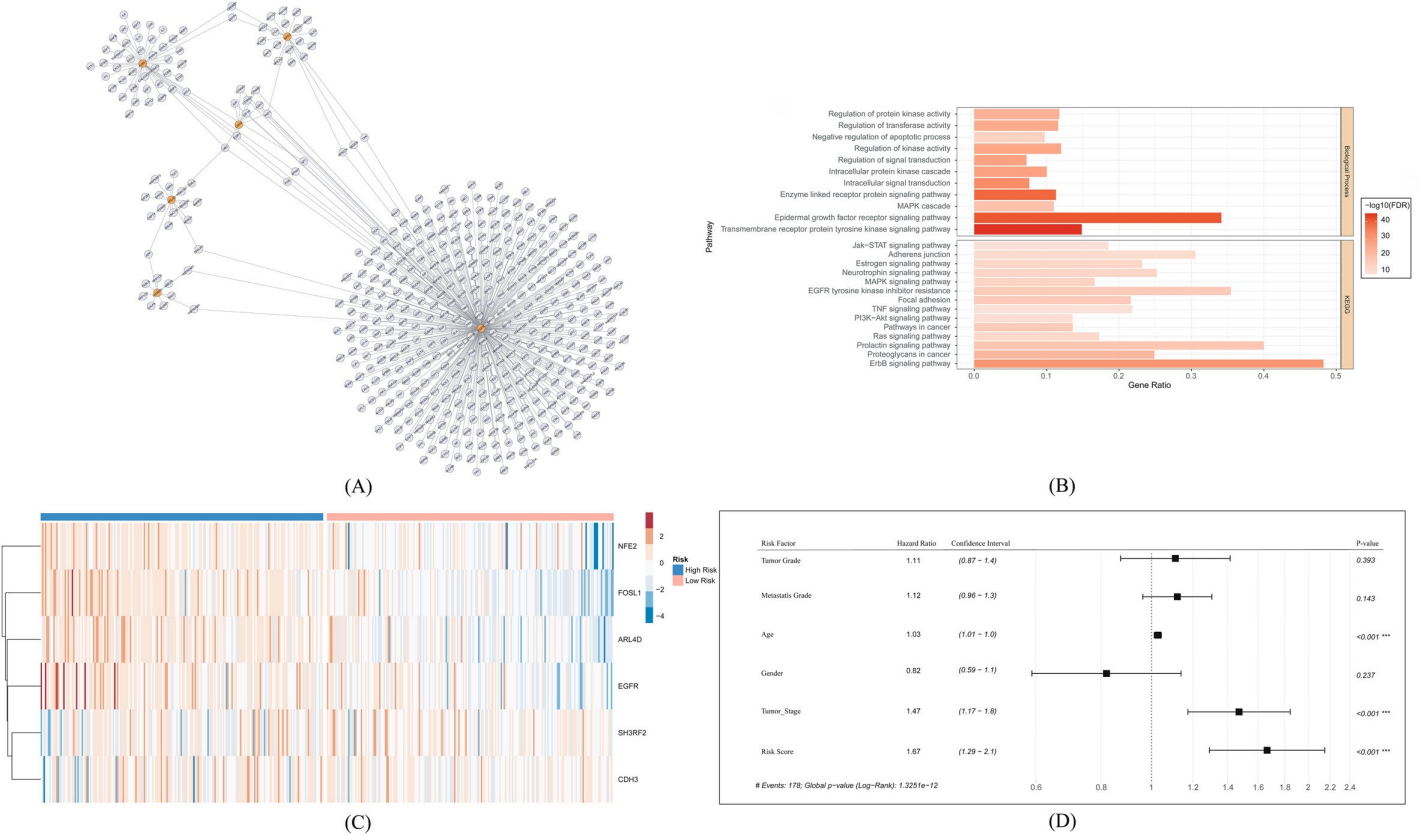
(**A**) Network analysis of the prognostic gene and hub gene identification. Orange color in the network indicated the hub genes. (**B**) Enrichment analysis of the prognostic genes where the color gradient was set based on the -log10(p-value). (**C**) Expression observation of 6 signature genes. (**D**) Multivariate analysis using clinical factors.

16 genes had significant findings from cox regression survival analysis (Supplementary Table 3). Among 16 genes 8 genes including SERPINB7 (Serpin family B member 7), FOSL1, ARL4D, NFE2, MBOAT2 (Membrane bound O-acyltransferase domain containing 2), ALDH1L2 (Aldehyde dehydrogenase 1 family member L2), TCHHL1 (Trichohyalin like 1), and EGFR, had a hazard ratio (HR) value greater than 1 which indicated that they can act as a hazardous gene in bladder cancer, while the rest had HR value less than 1 predicting their role in the survival of the cancer patients^60^. Following the analysis, 16 genes were then subjected to the network (Fig. 6A) and pathway analyses, which revealed that these genes are involved in several cancer-related pathways including Jak-stat signaling, MAPK cascade, epidermal growth factor signaling, TNF signaling pathways, and many others (Fig. 6B). The network analysis revealed 6 hub genes including EGFR, ARL4D, CDH3, SH3RF2, NFE2, and FOSL1. These 6 hub genes were found in the center of large networks and therefore, may serve as significant prognostic genes (Fig. 6A). The risk scores for each sample were calculated based on the expression of these 6 genes, and the risk score cutoff values were used to categorize all BLCA patients into high- and low-risk groups. The expression profile of the genes was visualized in a heatmap (Fig. 6C).

### Multivariate analysis for the final prognostic genes using clinical parameters

Multivariate analysis was performed to confirm the prognostic capability of the six-signature gene set and the independence of other clinical characteristics. For this, several co-variables include age, gender, tumor grade, metastasis grade, tumor stage, and risk score. A risk score of the signature gene set was found to be a potent and independent factor in the TCGA bladder cancer populations after incorporating additional clinical factors to modify the multivariate analyses (HR = 1.67, 95% CI 1.29–2.1, *P* < 0.001) (Fig. 6D).

### Survival analysis of risk groups

**Figure 7:**
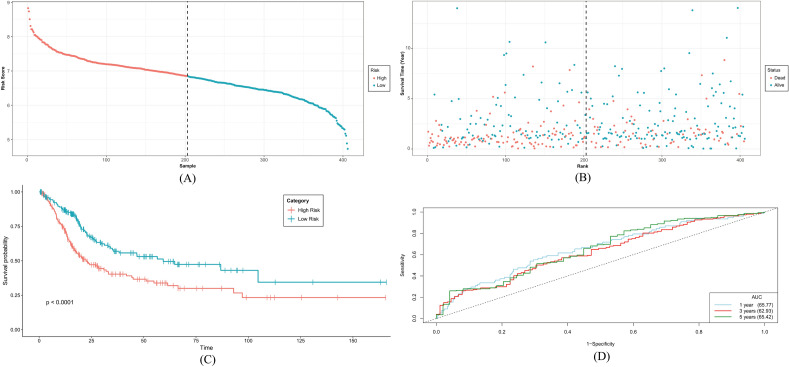
(**A**) Rank score and (**B**) survival status observation between high and low-risk groups. Based on the risk ratings of a set of six gene signatures, Kaplan–Meier OS curves were created for high and low-risk groups. (**C**) Overall survival plot demonstrates the difference between the two groups. The Log-rank test was used for the statistical analysis. The ROC analysis was carried out using the R program TIMEROC, as shown in (**D**). The analysis's False Positive Rate (AUC) was negligible, which is a strong indication of how well the risk score performed in terms of predicting OS for 1 year, 3 years, and 5 years.

The percentage of patients who died increased as the risk score increased (Fig. 7A,B). The chi-square test value between survival status and risk status was 13.663 with a *p*-value of 0.0002187 indicating a significant association between the two variables. Significant disparities between the High and Low-risk categories were observed through survival analysis and the ROC curve also validated the significance of survival analysis (Fig. 7C). The AUC value displayed satisfactory results indicating the risk model had high discriminatory power and it can accurately distinguish between patients who will survive and those who will not survive for 1 year, 3 years, and 5 years (Fig. 7D).

### Nomogram model generation

To predict the overall survival of BLCA patients, a nomogram was created by integrating the six-gene signature risk score with clinical variables such as age, gender, metastatic grade, tumor grade, and cancer stage. Nomogram results were consistent across all clinical variables. A declining survival probability was visible with the points at 1 year, 3 years, and 5 years (Supplementary Fig. 6A). Moreover, the calibration plot showed the best predictive accuracy, with the anticipated survival rate being about equal to the actual survival rate (Supplementary Fig. 6B–D). The nomogram combining the five-gene signature, grade, age, and gender may improve the prognosis prediction accuracy for BLCA patients.

### Performance evaluation and validation of the signature model

**Figure 8:**
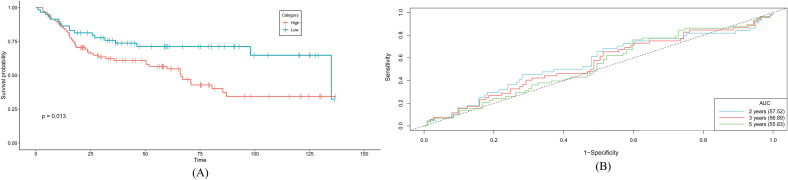
Validation of the risk model using external dataset by observing (**A**) overall survival and (**B**) roc curves between two risk groups. P-value was calculated to define the significance on the analysis.

An independent dataset GSE13507 was used for validating the risk model and survival analysis. Around 165 primary bladder cancer patients were selected with their survival data and divided into high and low-risk groups based on the mean value of the risk scores. The survival analysis was performed between two groups and the result showed higher risk group had lower survivability than the low-risk group which validates the findings of this study (Fig. 8A). The ROC curves were generated for 2 years, 3 years, and 5 years of overall survival and all the AUC values were above the baseline (AUC > 50%) (Fig. 8B). Also, the AUC curves were nearly parallel with identical shapes, indicating that the model's predictive performance remains consistent and stable over the period of measurement.

### Correlation analysis of the signature genes with interleukins and immune cell infiltration

To further evaluate the impact of hub genes, a correlation study between the expression of signature genes and IL-6 and IL-20 was carried out. The analysis showed that all genes were significantly positively associated with IL-6 except ARL4D (*P*-value > 0.05) and CDH3 (P-value > 0.05) and also significantly positively correlated with IL-20 (Supplementary Fig. 7A–L). Correlation between signature genes and immune filtration revealed that signature genes were shown to be favorably regulated with at least four levels of immune cell infiltration (Supplementary Fig. 8A–F), indicating the involvement of these genes in tolerating immune response. It was also observed that expression of the signature gene set was higher in high grade and also gradually increased in tumor stages (Supplementary Fig. 9) indicating the impact of the signature gene set in the development of high tumor grade and stage.

### Impact of mutations on signature genes

The mutational status between the two subtypes revealed that there are no driver missense mutations in the subtypes. Only NFE2 had a driver truncating mutation in subtype 1 (Supplementary Fig. 10A–F). These results indicated that there may be no association of mutations with expression patterns of these signature genes in both subtypes. This indeed signified the presence of crucial factors other than mutation including epigenetic, post-trasncriptional modificaitons, and environmental influence. This observation makes it more crucial to study the genetics behind the characteristics of these genes in cancer progression.

### Single-cell observation and grade comparison of signature genes

**Figure 9:**
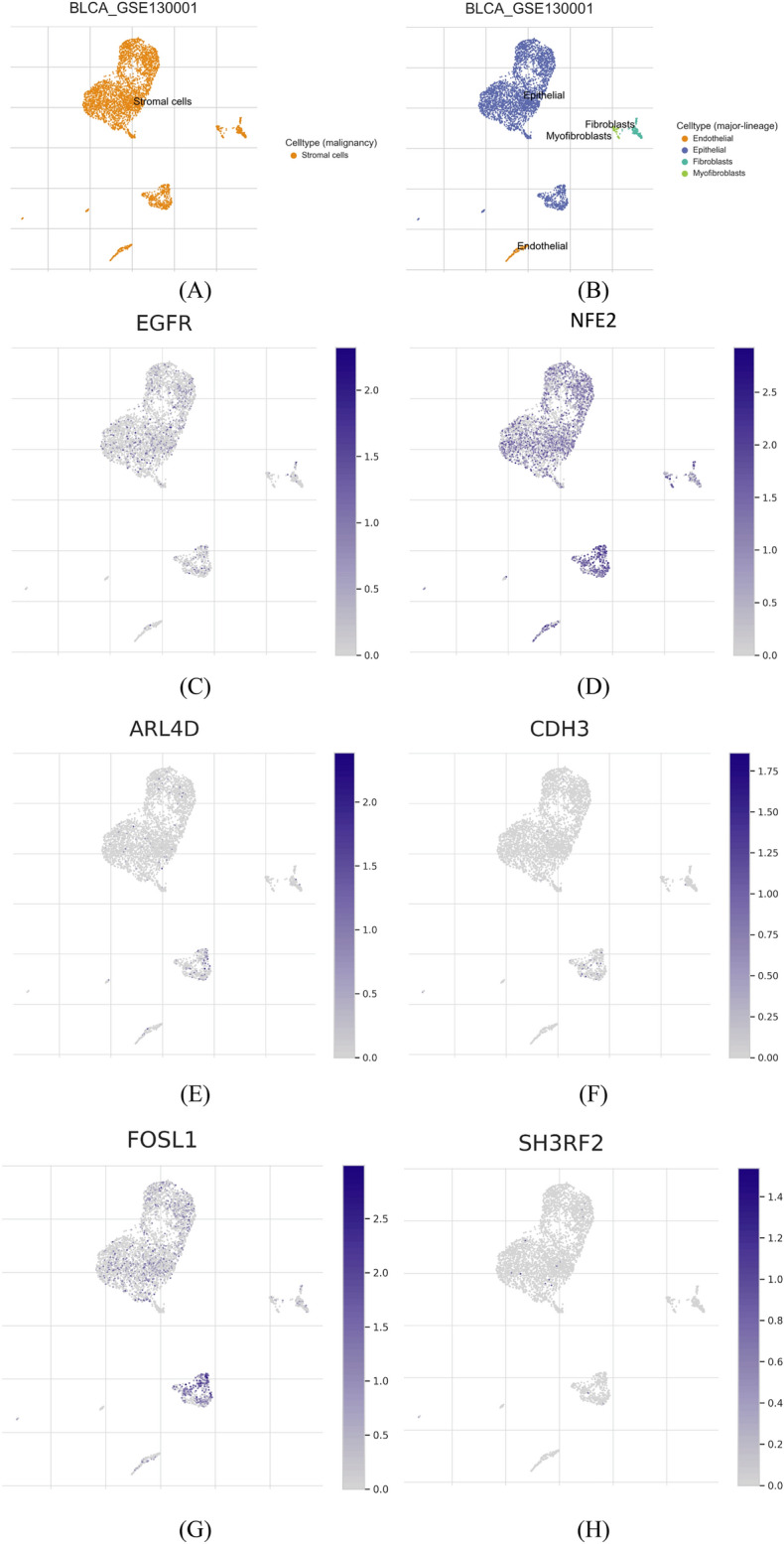
The single-cell RNA-seq analysis focuses on prognostic signature genes. (**A**) Utilizing the UMAP method, the distribution of cells in tumor microenvironment was visualized, and (**B**) the visualization of malignant stromal cells tumor microenvironment. The mRNA expression levels of (**C**) EGFR, (**D**) NFE2, (**E**) ARL4D, (**F**) CDH3, (**G**) FOSL1 and (**H**) SH3RF2, were examined across distinct cellular populations in tumor microenvironment.

**Figure 10:**
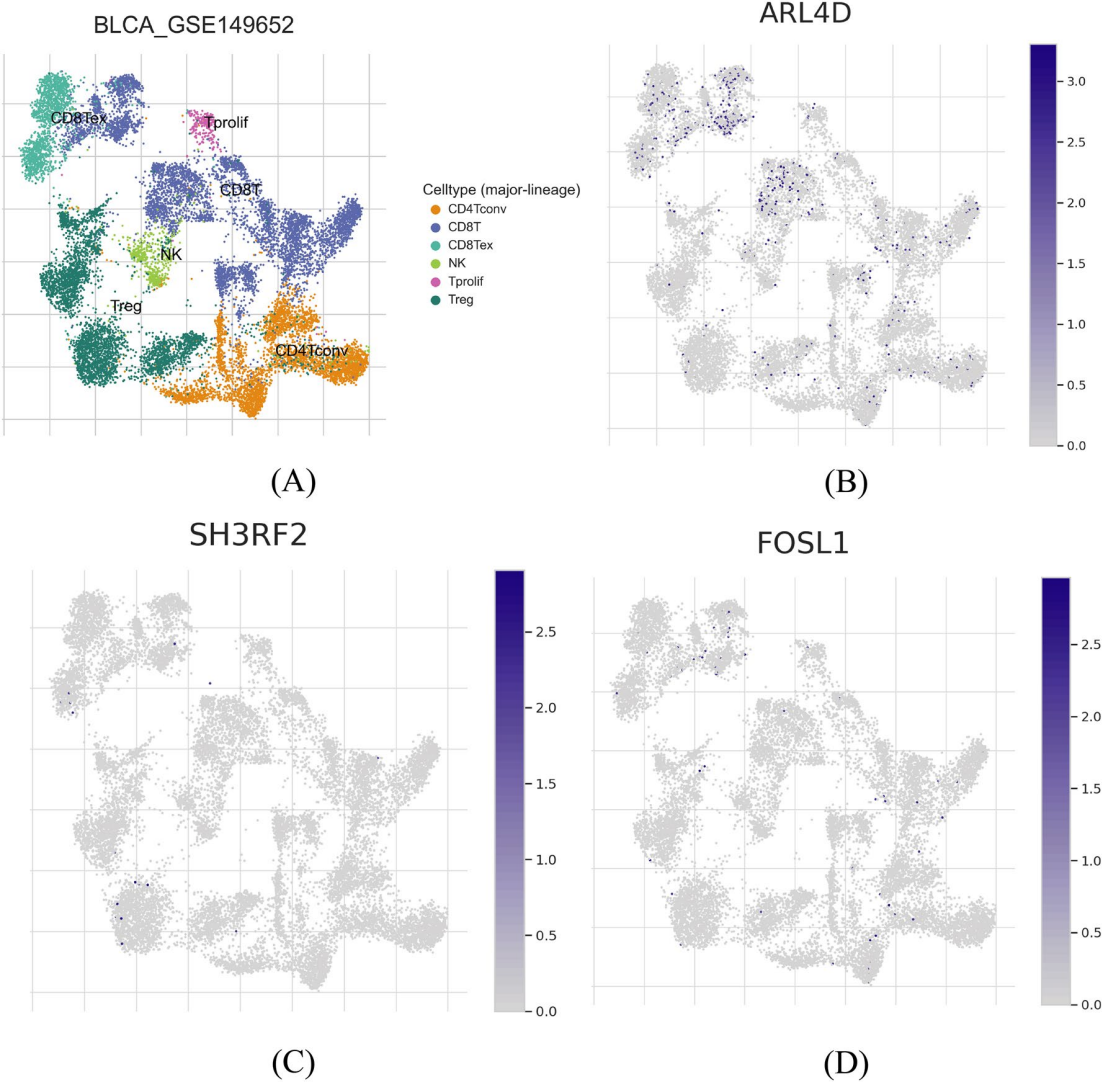
The single-cell RNA-seq analysis focuses on prognostic signature genes. (**A**) Utilizing the UMAP method, the distribution of Tumor-Infiltrating Cells (TICs) was visualized. The mRNA expression levels of (**B**) ARL4D, (**C**) SH3RF2, and (**D**) FOSL1, were examined across distinct TIC populations.

The analysis revealed most cells in tumor microenvironment including stromal cell expressed EGFR, NFE2, and FSL1 genes while a lower number of cells expressed CDH3, ARL4D, and SH3RF2 (Fig. 9). The expression of these genes in tumor microenvironment by cells that promote cancer progression indicated the the potential involvement of these genes in bladder cnacer development. In addition, ARL4D, SH3RF2, and FOSL1 were found to be expressed in tumor immune cells (TICs) (Fig. 10). Expression comparison between high and low grade patients for the signature genes showed that the signature genes tends to have a higher expression in high grade patients depicting the significance of these genes in the development of more advance form of cancer (Supplementary Fig. 11). EGFR, ARL4D, and NFE2 were significantly upregulated (*p*-value < 0.05) in high grade patients whereas the other were not significant but trend was higher in high grade patients.

## Discussion

Patients with BLCA have a high risk of metastasis and recurrence. Before immune checkpoint therapy, platinum chemotherapy has not been successful in treating metastatic BLCA throughout the last few decades^61–63^. With the rising acceptance of immunotherapy, emphasis has shifted to the creation of new biomarkers associated with tumor immune milieus that may be used to predict treatment efficacy and survival outcomes. Mechanistic investigations of cancer development now have a theoretical foundation thanks to the use of RNA-Seq and bioinformatic analysis of databases and allow researchers to simultaneously analyze the expression of thousands of genes across multiple samples^64^. This makes it possible to identify changes in gene expression that are associated with different experimental conditions or disease states. The purpose of this research was to identify a signature gene set that might be used as a biomarker for BLCA patients by utilizing bulk methylation and RNA-Seq data from TCGA BLCA data.

There are currently few biomarkers that can forecast both clinical outcomes and the effectiveness of immunotherapy. Previously performed studies only considered a particular criteria for instance expression data, gene-specific approach, etc. to propose biomarkers^65,66^. Some research work proposed signature models with good performance but they all followed a different approach to create such gene sets and did not consider hallmark pathways along with other gentic fators^67,68^. The study aimed to refine the prognostic accuracy and therapeutic strategies for BLCA patients through the integration of multi-omics data and the application of advanced bioinformatics methodologies. The novelty of the research lies in its comprehensive integration of RNASeq, methylation, and clinical data from TCGA, offering a holistic perspective on BLCA that considers the intricate interplay between genetic alterations and clinical outcomes. Using integrated methylation and transcriptomic data, along with clinical features a prediction model with six signature genes was created in this work which was also validated using an external dataset. Various analyses were performed to support the identification of the signature gene set including, multivariate analysis, survival analysis, correlation analysis, expression observation in tumor grade and stage, etc. This model can predict a patient's survivability and prognosis.

Initially, methylation data were extracted from TCGA and differential analysis was performed between normal and cancer patients performed. Following the analysis, prognostic genes were identified by using univariate cox regression analysis which revealed 793 genes. Hallmark pathways including the KRAS signaling pathway^69^, epithelial-mesenchymal transition^70^, DNA repair^71^, glycolysis^72^, signaling^73^, and a few other pathways-related genes were then screened through the prognostic genes and 71 hallmark pathway genes were found to be differentially methylated from the process which was involved in the survival of the cancer patients and used for NMF clustering of the BLCA patients by exploiting the expression data of these 71 genes.

To determine the ideal number of subtypes for the samples, numerous test runs with a rank of 2:6 were performed in this case. The cophenetic coefficient began to decrease after 2, indicating that 2 would be the ideal number of subtypes and the consensus matrix supported the clustering into 2 subtypes (Fig. 3A,B). To further validate the clustering, PCA analysis was performed and the plot was clearly divided into two portions. Immune checkpoint genes are important in regulating the immune system and preventing excessive immune responses that can lead to tissue damage therefore, immune checkpoint gene expression was observed between two subtypes (Fig. 5A). The higher expression of immune checkpoint genes in subtype 2 suggested that this subtype may have a stronger immune response and a greater ability to recognize and attack tumor cells. This could potentially make subtype 2 more responsive to immune-based therapies that target these checkpoint proteins. On the other hand, the lower expression of these genes in subtype 1 suggested that this subtype may be less responsive to immune-based therapies, and may require different treatment approaches. The expression pattern of chemokines was also observed between two subtypes and was highly expressed in subtype 2 indicating a poor prognosis of subtype 2 (Fig. 5B). Furthermore, CXCL14 was found to be highly expressed in subtype 1 and studies suggested that this chemokine is responsible for better prognosis in prostate cancer which opens up a possibility for this chemokine to become an effective marker for bladder cancer. The link between CD4 and CD8 cells is still unclear, despite changes in the numbers of neutrophils, NK cells, macrophages, dendritic cells, and mast cells in BLCA^74^. Several studies showed that CD8 is responsible for a better prognosis of bladder cancer. Throughout the work, it was found that individuals with subtype 1 who had a better prognosis had a higher CD8 cell count^75^. On the other hand, a worse prognosis has been associated with higher CD4 cell counts and CD3/CD4 ratios, which was corroborated in this study for subtype 2^76^.

Differential expression and gene ontology analysis revealed that genes upregulated in subtype 1 were involved in biosynthesis-related pathways whereas genes in subtype 2 were involved in immune response-related pathways. It was also observed that individuals from subtype 2 were enriched in various cancer-related pathways including the JAK-Stat signaling pathway, TNF signaling pathway, and NF-kB signaling pathway indicating the worst survivability of subtype 2 compared to subtype 1. Subtype 1 showed a high enrichment score for cytochrome P450-related pathways, which might be explained by the fact that these patients' improved prognoses led to better drug metabolism responses to anticancer therapy. Univariate analysis and subsequent lasso regression and network analysis discovered 6 hub genes and considered as the most significant prognostic gene set which were EGFR, FOSL1, NFE2, ARL4D, SH3RF2, and CDH3 (Fig. 6A).

EGFR is positively associated with poor outcomes in bladder cancer. It also promotes cancer growth, progression, and metastasis of various cancers^77^. FOSL1 or FOS Like 1, AP-1 Transcription Factor Subunit is a leucine zipper protein that regulates tumor cell proliferation and survival in cancer. This protein was found to be involved in controlling the motility of bladder cancer cells through upregulating receptor tyrosine kinase AXL^78^. NFE2 is mostly involved in the regulation of maturation and biogenesis of platelets, cellular detoxification, and drug influx/efflux and was found to be involved in bone metastasis by promoting the WNT signaling pathway. This was found to be associated with poor prognosis and chemotherapy resistance in bladder cancer^79^. ARL family proteins including ARL4D, are involved in cancer cell migration, invasion, and proliferation^80^. Cancer cells are successfully kept from undergoing apoptosis when SH3RF2 is expressed ectopically because it promotes cell motility, colony formation, and tumor development in vivo^81^. The ability of cells to adhere to the extracellular matrix and other cells is mediated by the cell adhesion protein CDH3. It also regulates tissue morphology and is found to be highly expressed in different malignancies including bladder cancer^82^.

Based on the expression pattern and survival coefficients of these 6 prognostic genes, the risk score model was generated and risk scores were calculated for each TCGA BLCA patient. Multivariate analysis disclosed that risk score can independently predict the survivability of bladder cancer patients which strengthens the potential of the risk model. A Kaplan–Meier survival analysis between the high-risk and low-risk groups was performed to corroborate these findings, and the results showed that the high-risk groups had a poorer prognosis rate with significant log-rank test values.

Based on the clinical data and risk score, a nomogram model was developed. It depicts a mathematical likelihood of a clinical event from a statistical prediction model. It can be used to combine various prognostic factors, such as tumor stage, age, tumor markers, and treatment modalities, to estimate the likelihood of certain outcomes. This clinical prediction nomogram may help create personalized treatment strategies for BLCA patients by offering each patient a predicted result.

Using the independent dataset GSE13507, the risk score for six genes was assessed. High-risk groups had significantly lower values in the survival plot. Nonetheless, the independent dataset's AUC curve produced values above the baseline, validating the survival analysis's findings (Fig. 8). However, the results need to be further verified because of the data variation and differences in data distribution in the independent dataset GSE13507 since it was generated from the Korean population, whereas the data used in this study came from the American population. In addition, the TCGA BLCA data considered a wide variety of data types such as geographical locations, gender, age, biopsy location, age, race, smoking, tumor grade, metastasis, etc. whereas the independent dataset considered only a few factors.

To further strengthen prediction for 6 signature genes, the association between interleukins and immune cell filtration was observed. IL-6 promotes bladder cancer progression through AKT and STAT3 activations which ultimately lead to epithelial-mesenchymal transition and angiogenesis. Interleukin-20 induces the up-regulation of p21(WAF1) protein expression, which in turn causes nuclear factor (NF-B) to activate by promoting the migration of bladder cancer cells via ERK-mediated MMP-9 protein production. The correlation analysis revealed that the six signature genes were positively correlated with IL-6 and IL-20 indicating the dysregulation of these genes may hamper the regulation of IL-6 and IL-20 and may cause cancer progression. It was also found to be positively correlated with immune filtration cells including CD8 + , CD4 + , macrophage, and others. This implied that the expression of signature genes may react significantly in response to immune therapy. The signiature genes were also found to be expressed in tumor microenvironment by cells that promote cancer progression and expressed in immune-related genes which may provide valuable insights about immune therapy. The elevated expression levels of these genes in high-grade patients indicate their importance in the development of aggressive cancer forms and their relevance to prognosis.

It is essential to conduct proteomic investigations and analyze miRNAs that regulate mRNA translation^83^. By combining proteomic techniques with miRNA analysis, it is possible to gain valuable insights into the complex regulatory mechanisms controlling protein expression in cells. This integrated approach can provide a comprehensive understanding of how miRNAs influence the translation of specific mRNAs, leading to a more comprehensive understanding of cellular processes and potential therapeutic targets^84^. Integration of miRNA-lncRNA interactions using extensive machine learning model^85^ relating the signature genes may refine the molecular landscape, contributing to a more comprehensive and clinically relevant characterization of bladder cancer. Utilizing predictive models such as, DMFGAM which considers both molecular fingerprint and molecular graph features, can aid in prioritizing candidate biomarkers for further validation and experimental exploration^86^. Moreover, consideration of metabolite-disease associations through utilizing deep learning model, such as GCNAT^87^, will enhance the clinical relevance of the identified gene signature and underscores the multifaceted nature of molecular interactions in bladder carcinoma. Establishing intricate relationship networks in the context of signature genes with diseases may provide novel strategy to study the disease mechanisms and many deep learning approaches including, NSCGRN and MPCLCDA, are being developed that consider the biological data sources to understand the association with disease progression^88,89^. However, due to data availability, scope limitations, and resource constraints, these analyses weren’t included in the study but rather provided necessary insights based on which a comprehensive study can be designed to improve the robustness of the biomarkers proposed in this study in bladder cancer management.

The six-gene signature showed promising results as a robust prognostic biomarker, facilitating effective risk stratification and personalized treatment decisions in the context of bladder carcinoma. Its potential applications extend to informing clinical trial design, monitoring treatment efficacy, and identifying therapeutic targets. However, several challenges such as practical implementation, ethical considerations, disease heterogeneity, and predicting treatment responses need careful consideration before establishing the signature geneset as a viable biomarker. Ordinary Differential Equations (ODE)-based theoretical modeling studies on signature gene signaling networks could provide a more comprehensive understanding of regulatory mechanisms and potential therapeutic targets in bladder cancer^90^. In addition, Applying the insights from phase separation mechanisms and knowledge of intracellular organization to the intricate molecular landscapes of cancer cells can enhance the interpretation of biomolecular interactions, spatial patterns, and regulatory mechanisms, thereby contributing to the depth and applicability of your prognostic signature study in the context of bladder carcinoma^91^. As this study unveils the promising six-gene signature as a prognostic biomarker in bladder carcinoma, the integration of theoretical modeling studies further emphasizes the translational implications. While further validation and collaborative efforts are essential, successful implementation holds the potential to significantly enhance bladder cancer management, ultimately improving patient outcomes. Addressing these challenges will be pivotal in advancing more precise diagnostics and tailored interventions for bladder carcinoma.

In this study, a distinct signature gene collection based on methylation and transcriptomic data was discovered. Six genes were shown to be significantly associated with a patient's survival from bladder cancer. Some limits still need to be looked upon despite the unique insights that this study has provided. With a bigger sample size, the findings of this retrospective investigation would be more credible.

## Conclusion

In summary, the six signature genes EGFR, FOSL1, NFE2, ARL4D, SH3RF2, and CDH3 may serve as possible biomarkers for BLCA patients' prognoses. This signature collection showed strong prediction performance in both the training and validation cohorts and might be used to more precisely identify prognostic risk in bladder cancer patients. This work may offer insights for additional research into the biological processes, clinical diagnosis, and treatment approaches of BLCA related to these genes. It may also lead to the development of targeted therapies, exploration of epigenetic treatments, and integration of other omics data for a more comprehensive understanding of bladder cancer.

### Supplementary Information

Supplementary Figure 1.

Supplementary Figure 2.

Supplementary Figure 3.

Supplementary Figure 4.

Supplementary Figure 5.

Supplementary Figure 6.

Supplementary Figure 7.

Supplementary Figure 8.

Supplementary Figure 9.

Supplementary Figure 10.

Supplementary Figure 11.

Supplementary Table 1.

Supplementary Table 2.

Supplementary Table 3.

Supplementary Legends.

## Data Availability

The datasets utilized in this study are available in TCGA (https://portal.gdc.cancer.gov/) and GEO repository (https://www.ncbi.nlm.nih.gov/geo/).
