# Supplementary figures and images for: Identification and validation of prognostic signature genes of bladder cancer by integrating methylation and transcriptomic analysis
Source: Sci Rep. 2024 Jan 3;14:368. doi: 10.1038/s41598-023-50740-x (PMC10764961; doi:10.1038/s41598-023-50740-x)

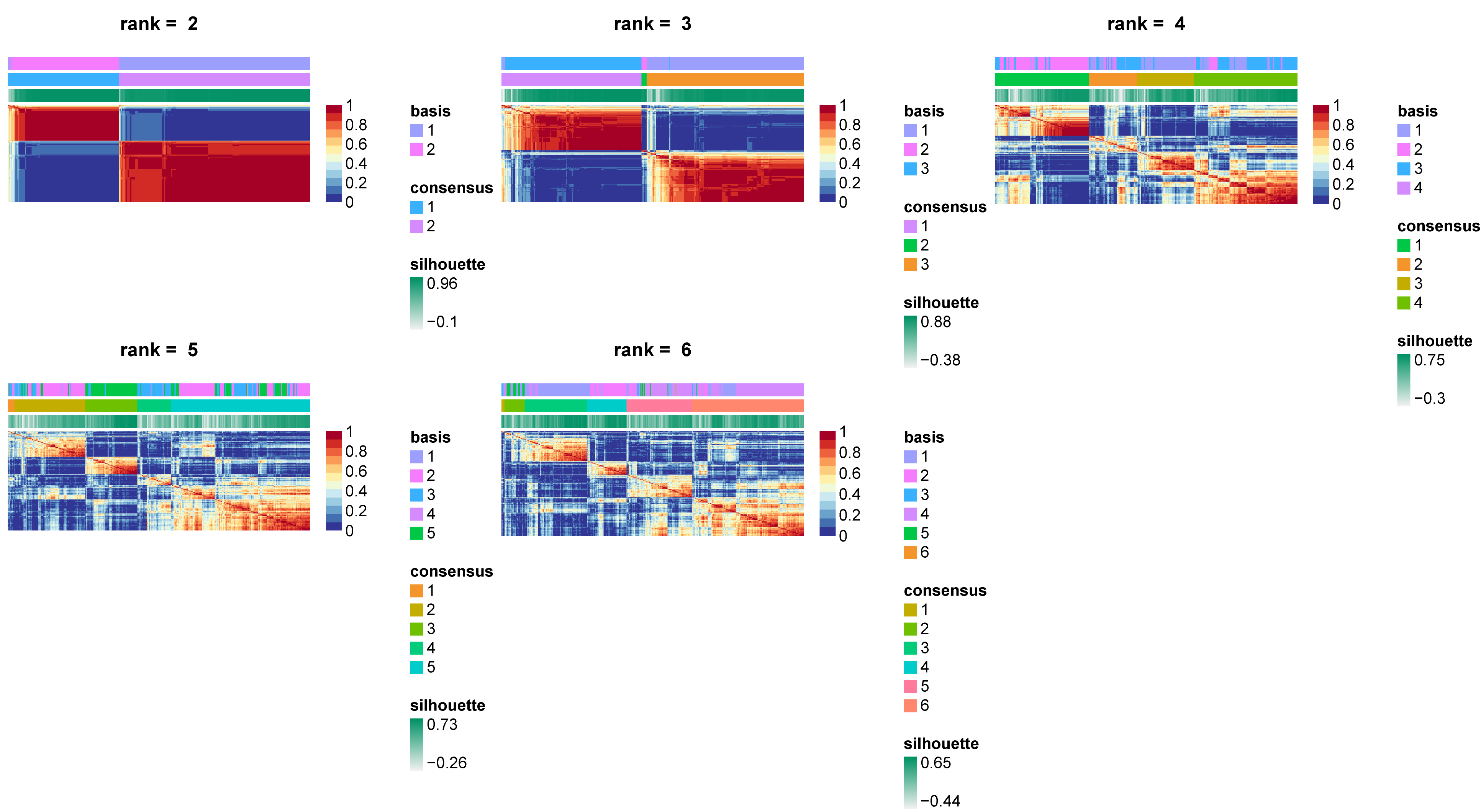

Supplement: Supplementary file 1 — Supplementary Figure 1. [file 41598_2023_50740_MOESM1_ESM.tif]

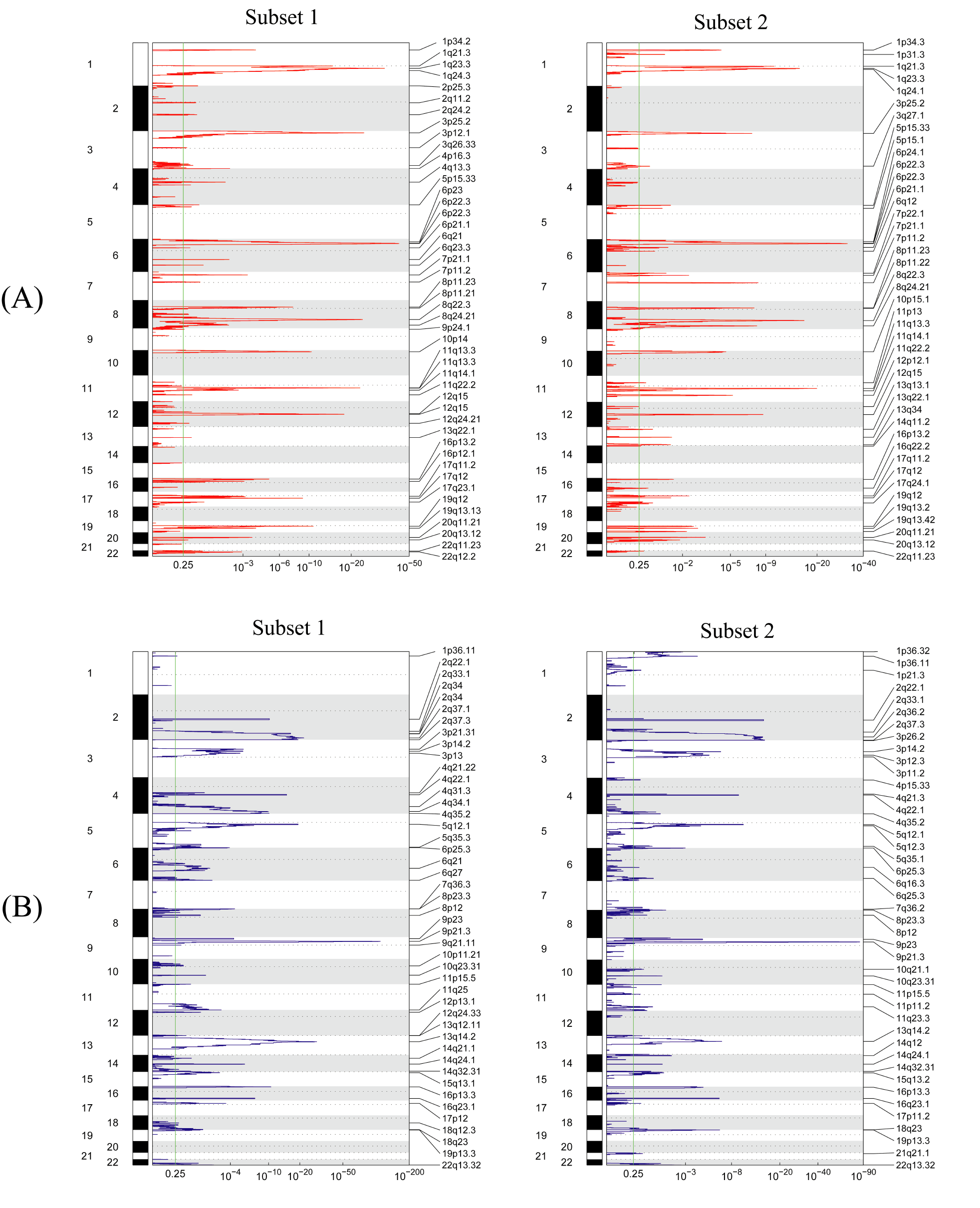

Supplement: Supplementary file 2 — Supplementary Figure 2. [file 41598_2023_50740_MOESM2_ESM.tif]

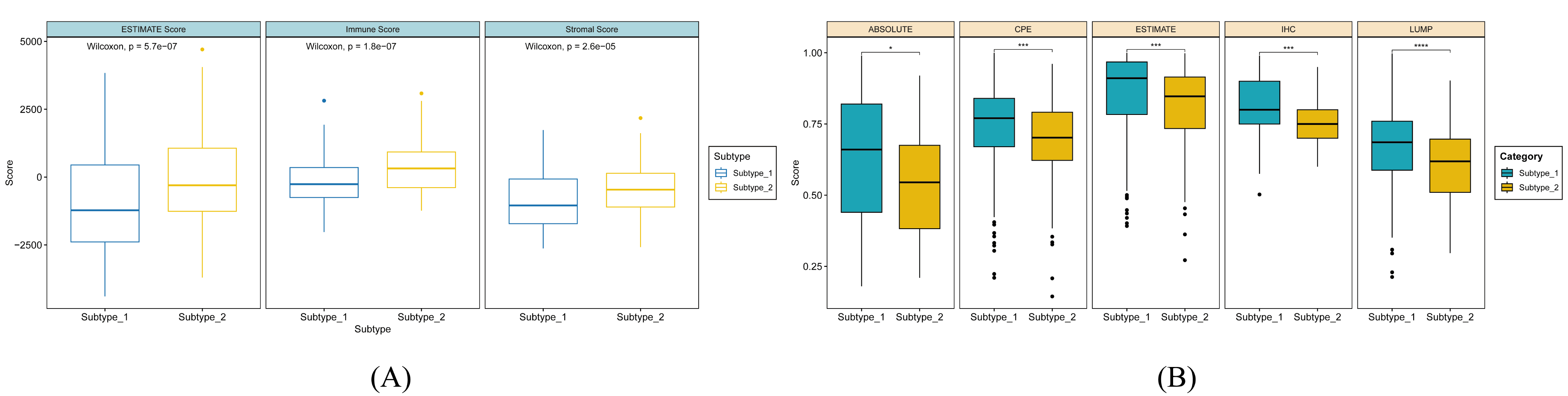

Supplement: Supplementary file 3 — Supplementary Figure 3. [file 41598_2023_50740_MOESM3_ESM.tif]

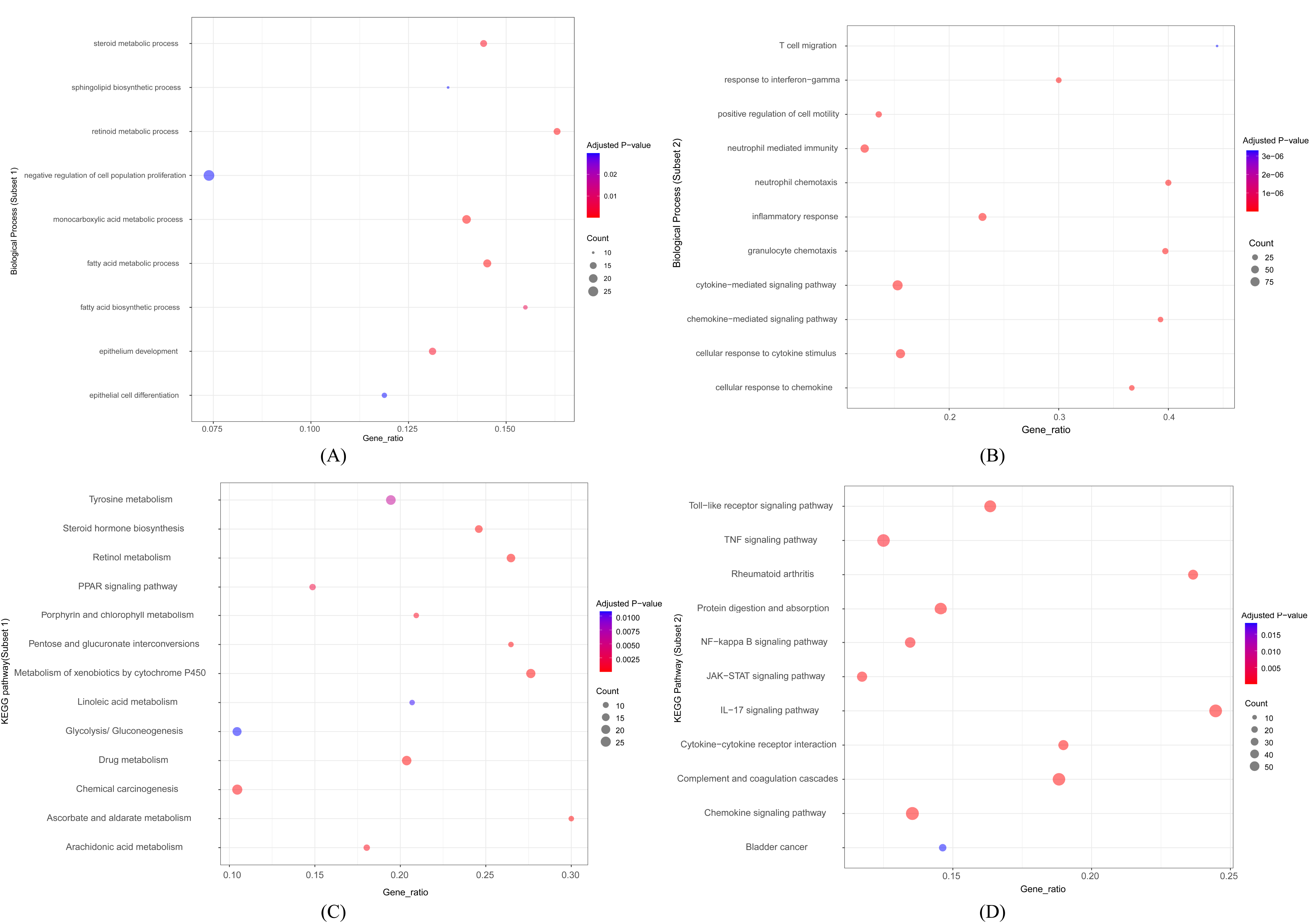

Supplement: Supplementary file 4 — Supplementary Figure 4. [file 41598_2023_50740_MOESM4_ESM.tif]

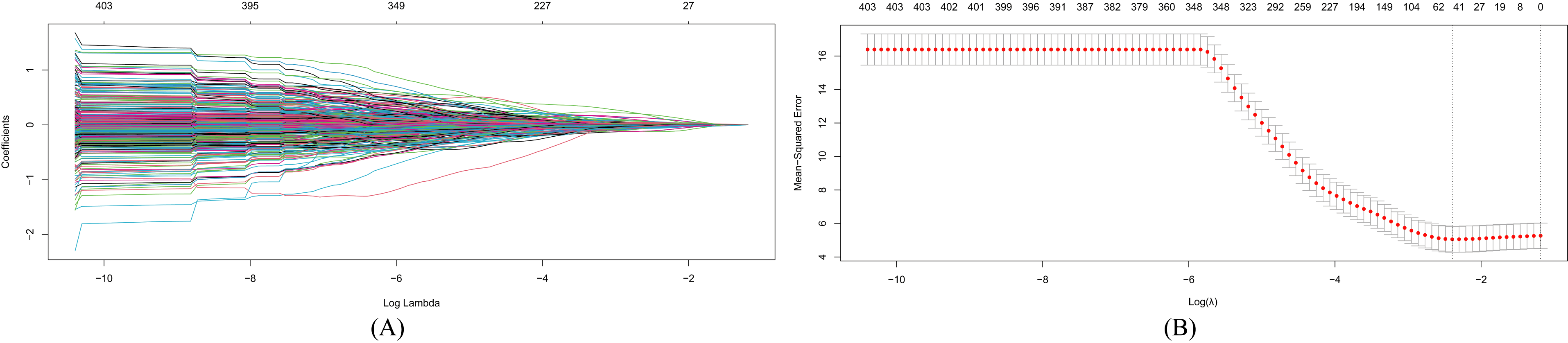

Supplement: Supplementary file 5 — Supplementary Figure 5. [file 41598_2023_50740_MOESM5_ESM.tif]

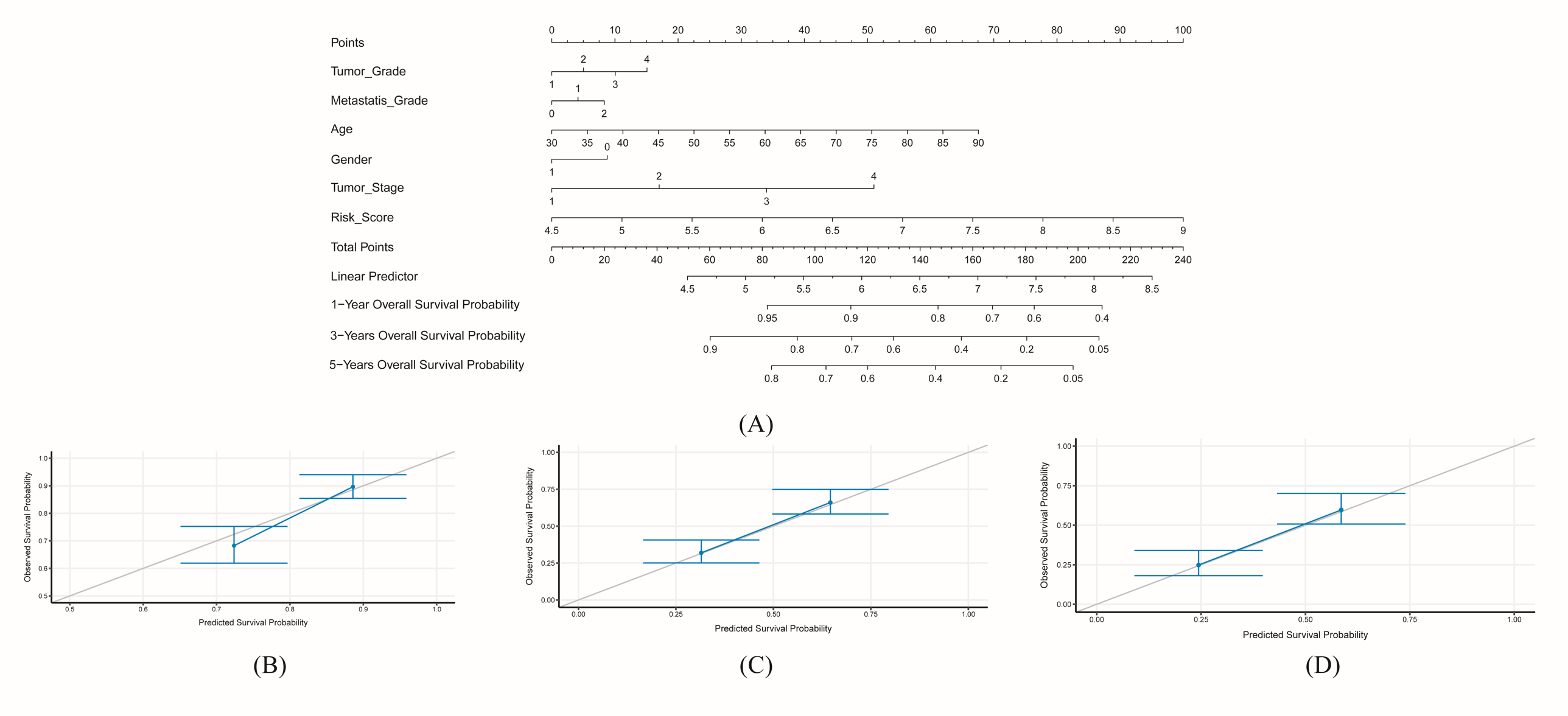

Supplement: Supplementary file 6 — Supplementary Figure 6. [file 41598_2023_50740_MOESM6_ESM.tif]

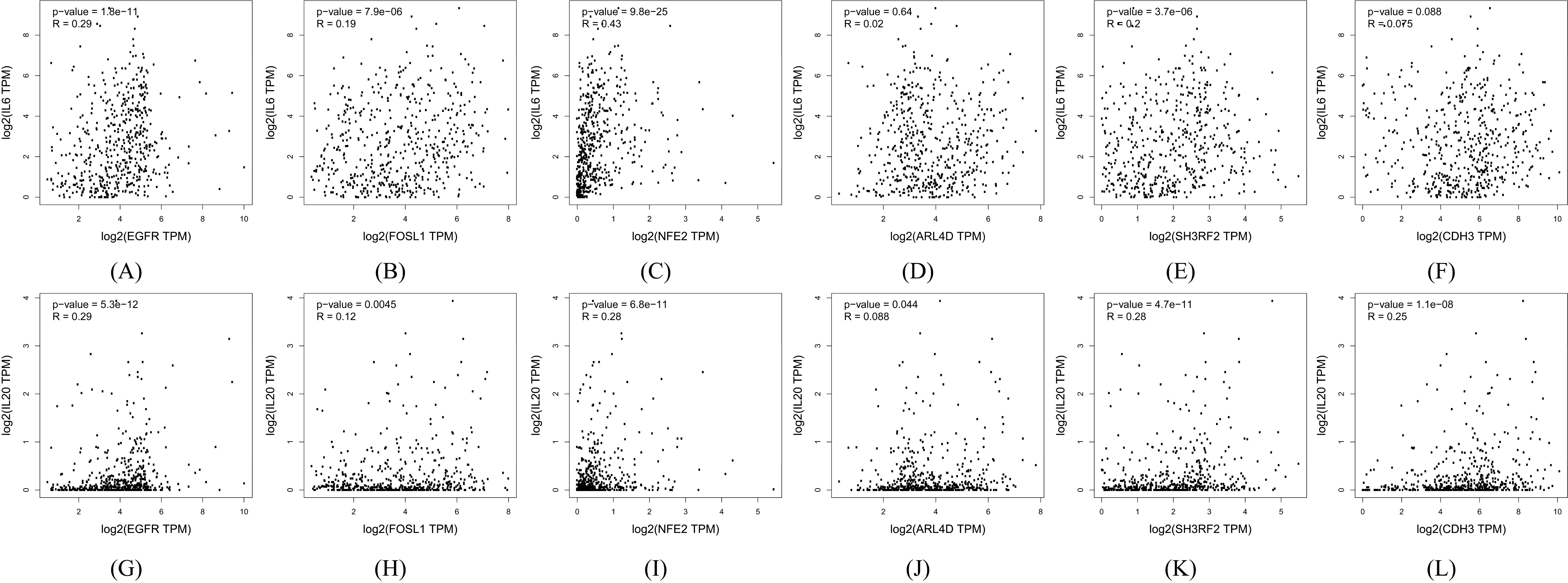

Supplement: Supplementary file 7 — Supplementary Figure 7. [file 41598_2023_50740_MOESM7_ESM.tif]

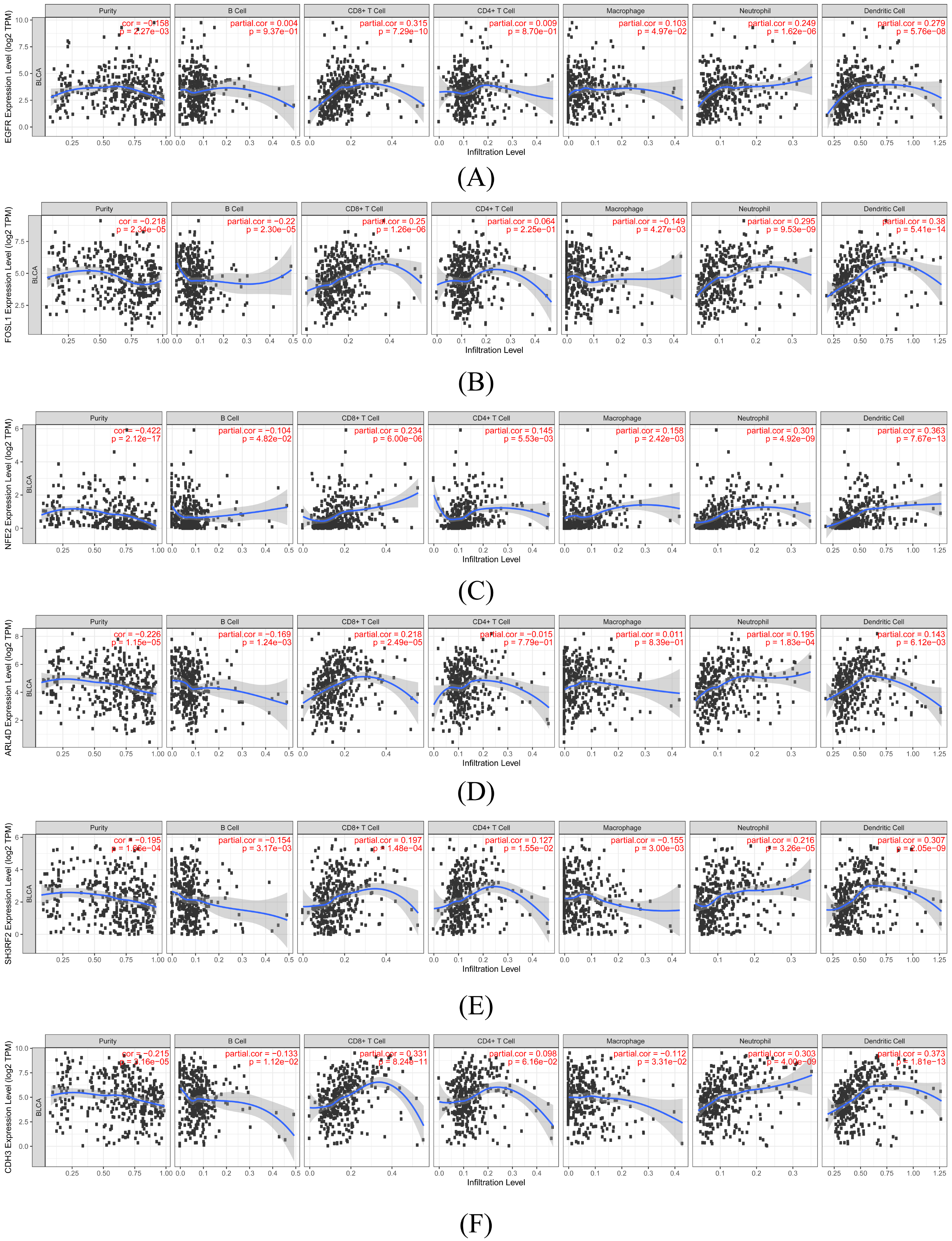

Supplement: Supplementary file 8 — Supplementary Figure 8. [file 41598_2023_50740_MOESM8_ESM.tif]

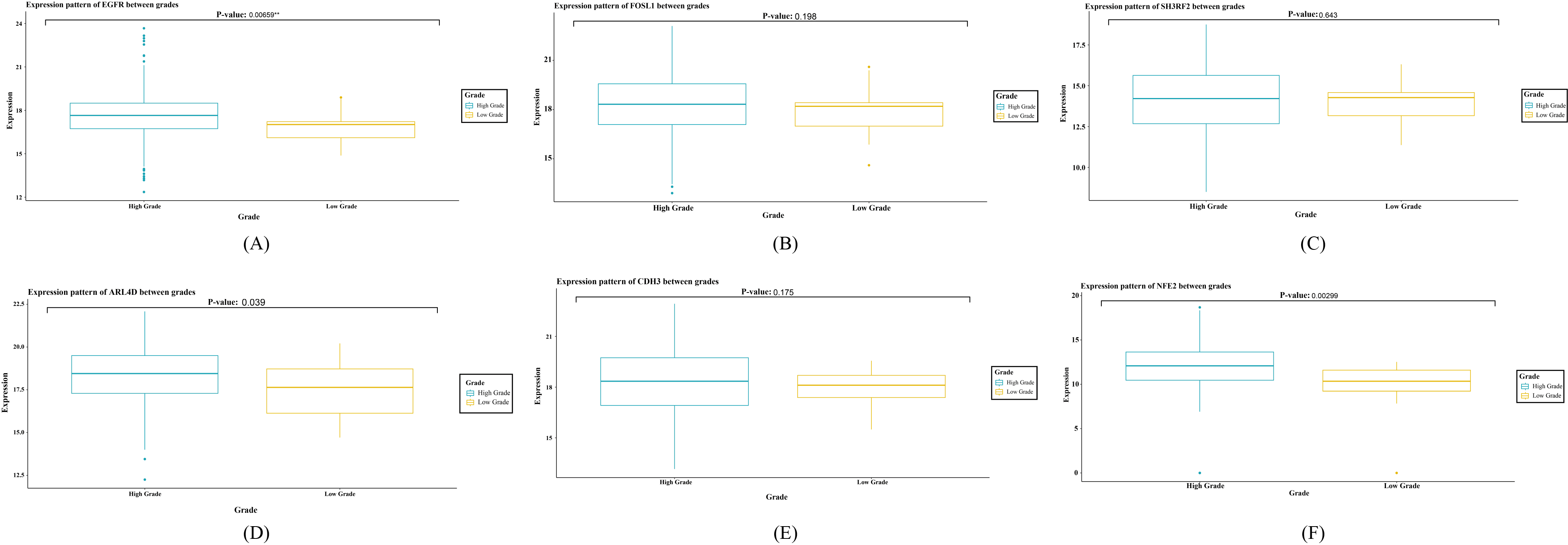

Supplement: Supplementary file 11 — Supplementary Figure 11. [file 41598_2023_50740_MOESM11_ESM.tif]
