# Supplementary material for: Identification and validation of prognostic signature genes of bladder cancer by integrating methylation and transcriptomic analysis
Source: Sci Rep. 2024 Jan 3;14:368. doi: 10.1038/s41598-023-50740-x (PMC10764961; doi:10.1038/s41598-023-50740-x)
Supplement: Supplementary file 15 — Supplementary Legends. [file 41598_2023_50740_MOESM15_ESM.docx]

**Supplementary Material Caption**

**Supplementary Table 1.** Tools and algorithms used in this study with proper function and parameter.

**Supplementary Table 2.** CIBERSORT result between two subtypes including p-value from Wilcoxon signed rank test.

**Supplementary Table 3.** Cox regression analysis result of 16 significant genes.

**Supplementary Fig. 1.** Heatmap for all K ranks.

**Supplementary Fig. 2.** Chromosome amplification (A) and deletion (B) plots for subtype 1 and subtype 2.

**Supplementary Fig. 3.** Stromal score, immune score, and estimate score comparison between subtypes. The survival analysis of our study was validated by subtype 2, which demonstrated high levels of the stromal score, immune score, and ESTIMATE score suggesting a poorer prognosis (A). Tumor purity scores comparison between subtype 1 and subtype 2. (B) In all the approaches, the score for subtype 2 is lower than subtype 1, indicating a poorer prognosis in subtype 2 samples. To determine the P-value, the Wilcoxon rank test was used.

**Supplementary Fig. 4.** Functional enrichment analysis between subtype 1 and subtype 2. Biological process observation for upregulated genes of (A) subtype 1 and (B) subtype 2. KEGG pathway analysis between (C) subtype 1 and (D) subtype 2.

**Supplementary Fig. 5.** Lasso regression model observation of DEGs using (A) survival time and (B) minimum lambda value 0.09114313. The model provided 50 significant genes.

**Supplementary Fig. 6.** The creation of a nomogram including clinical traits and subtype-specific markers for predicting survival (A). Nomogram calibration plots showed the observed and expected probabilities of one-, three-, and five-year OS (B-D).

**Supplementary Fig. 7.** Correlation analysis of signature gene sets with IL-6 (A-F) and IL-20 (G-L).

**Supplementary Fig. 8.** Correlation between hub genes EGFR (A), FOSL1 (B), NFE2 (C), ARL4D (D), SH3RF2 (E), and CDH3 (F) levels and immune cell infiltration in bladder cancer tissues. Each dot represents a sample in the TCGA cohort.

**Supplementary Fig. 9.** Expression pattern of the signature gene set in tumor grade (A-F) and tumor stage (G-L).

**Supplementary Fig. 10.** Mutational assessment of gene signatures in subtypes (A-F).

**Supplementary Fig. 11.** Expression comparison between high and low grade TCGA BLCA patients for (A) EGFR, (B) FOSL1, (C) SH3RF2, (D) ARL4D, (E) CDH3, and (F) NFE2. Independent T-test was performed for significance test.
